# Impact of Cervical Cancer on Quality of Life and Sexuality in Female Survivors

**DOI:** 10.3390/ijerph20043751

**Published:** 2023-02-20

**Authors:** Lucia Membrilla-Beltran, Diana Cardona, Laura Camara-Roca, Adrian Aparicio-Mota, Pablo Roman, Lola Rueda-Ruzafa

**Affiliations:** 1 Maternity Hospital Virgen de las Nieves, 18014 Granada, Spain; 2 Faculty of Health Sciences, Department of Nursing, Physiotherapy and Medicine, University of Almeria, 04120 Almeria, Spain; 3 Health Research Center, University of Almería, 04120 Almería, Spain; 4 Andalusian Public Foundation for Biomedical Research in Eastern Andalusia (FIBAO), University Hospital Torrecárdenas, 04009 Almería, Spain

**Keywords:** cancer survivors, sexual dysfunction, quality of life, orgasm, retrospective

## Abstract

Cervical cancer is the fourth most frequent cancer in women worldwide, and the 11th most frequent neoplasm in Spain. Despite the optimization of treatments and a 5-year survival rate of 70%, side effects and sequelae are described after treatment. The treatments have physical, psychological and sociocultural consequences that deteriorate the quality of life of patients. One of the sequelae that worries patients is the impairment of sexual function and satisfaction, considered a fundamental dimension of the human being. The aim of this study was to examine quality of life and sexual function and satisfaction among Spanish cervical cancer survivors. A retrospective case-control study was conducted between 2019 and 2022. The sample consisted of 66 patients who completed the Female Sexual Function Index, the Golombok Rust Sexual Satisfaction Inventory and European Organization for Research and Treatment of Cancer quality of life questionnaire. The control group consisted of women without cervical cancer and gynecological pathologies obtained using the so-called online virtual sampling method. The patient group consisted of women with cervical cancer who completed treatment. Cervical cancer survivors reported sexual dysfunction and impaired sexual satisfaction in almost half of the domains. Quality of life was also affected, with pain and fatigue being the most frequent symptoms in these patients. Our results indicate that there is dysfunction, sexual dissatisfaction and a lower level of quality of life in cervical cancer survivors than in healthy women without pathology.

## 1. Introduction

Cervical cancer is considered a public health problem in the 21st century, as it is estimated that more than 500,000 women worldwide suffer from it and around 300,000 have died from the disease. This cancer was the fourth most common cancer in women, after breast, colorectal and lung cancer, worldwide in 2018 [1]. It is expected that in the next 10 years, the premature mortality rate from cervical cancer can be reduced by one third [2]. Therefore, due to the increased survival of cervical cancer, it is necessary to improve the quality of life of these patients [3,4]. In this vein, sexual satisfaction, in which the cervix plays an important role in orgasm and sexual function, is a fundamental dimension of the human being and is recognized as a good indicator of quality of life [5].

Several studies have reported deficits in sexual function as well as poorer quality of life in these patients [6,7]. For example, the study by Harding and colleagues (2014) found that patients who had received radiotherapy had lower levels of sexual satisfaction than controls, scoring significantly lower on all dimensions of The Female Sexual Function Index (FSFI) [7]. The radiotherapy and chemotherapy are risk factors for sexual function. However, owing to these factors, patients with preserved ovaries tend to regain a satisfactory quality of sexual life after recovery from surgery [8]. Notably, worse sexual function is detected in the advanced stages of the disease, regardless of treatment [9].

Similar results were obtained in patients treated with neoadjuvant chemotherapy (NACT) and radical hysterectomy. These patients experience reduced sexual desire, lack of arousal and orgasm, decreased lubrication and sensation, premature menopause, loss of fertility, reduced vaginal elasticity, vaginal shortening, atrophy and stenosis, and cystitis, often leading to sexual dysfunction [3]. In addition, a qualitative study reports that cervical cancer patients have low libido due to cancer symptoms and chemotherapy side effects, which means that cervical cancer affects all aspects of women's health, including sexual function and physical wellbeing [10]. It has also been associated with symptoms related to anxiety and depression, as Park and colleagues (2007) found that cervical cancer survivors reported higher anxiety and worse sexual functioning compared to controls, as well as dyspareunia in women who received radiotherapy [11]. Regarding quality of life in cervical cancer survivors, a recent systematic review shows that some symptoms disappear in the first 3 months after completing treatment, whereas menopausal symptoms and sexual concerns persist after curative treatment [12]. 

In summary, sexuality is a dimension of quality of life affected in these patients, which correlates with depression. However, quality studies are scarce, and the results cannot be generalized [13]. In fact, several of these studies have limitations such as the use of non-standardized questionnaires or the use of a single questionnaire [14,15]. In other studies, although sexual satisfaction is assessed, it is interpreted as a subjective measure of sexual function rather than as a dimension in itself [16]. 

Therefore, considering the above mentioned and given its high incidence as well as the impact on their quality of life, it is necessary to measure the sexuality and quality of life of cervical cancer survivors. Thus, the aim of this study was to compare quality of life and sexual functioning and satisfaction between cervical cancer survivors and healthy women in a Spanish population using standardized questionnaires.

## 2. Materials and Methods

### 2.1. Study Design

A retrospective case–control study was performed between 2019 and 2022. The study was approved by the Research Ethics Committee of the Hospital Materno-Infantil Virgen de las Nieves in Granada (Spain) and complied with the Declaration of Helsinki. Participants did not receive any funding to conduct this research and informed consent was obtained from all subjects who participated in the study. 

The study included all cervical cancer survivors who completed treatment, were discharged between January 2019 and February 2022, and met the inclusion criteria. The study group was collected using the so-called online virtual sampling method. Data were collected following the Strengthening the Reporting of Observational Studies in Epidemiology (STROBE) guidelines to reduce any risk of bias [17]

### 2.2. Patient and Public Involvement Statement

The sample size was calculated with the Epidat software (V.4.2) (Epidemiology Service of the Dirección Xeral de Saúde Pública da Consellería de Sanidade (Xunta de Galicia, Galicia, Spain), with a power of 80%, a confidence level of 95%. Significant differences in the FSFI questionnaire were considered to be of 6.9 in the mean, and assuming a standard deviation of 9.6 for the experimental group and 9.9 for the control group. In addition, a minimum sample size of 33 women in each group was obtained. 

The patient group was recruited through the database provided by the Cervical Cancer Unit of patients treated at Hospital Materno-Infantil Virgen de las Nieves in Granada (Spain). The inclusion criteria for the group were as follows (1) women 18 years of age or older, (2) having a diagnosis of cervical cancer at different stages (I, II or III), (3) having been discharged from hospital and having completed cancer treatment at least three months before the start of the study (time expected for the appearance of medium and long-term side effects), (4) be able to read and write Spanish, (5) voluntarily agree to participate in the study in accordance with the Declaration of Helsinki, (6) sign the informed consent and (7) not be participating in another study that could infer the results obtained. Patients with the following characteristics were not included in the study: (1) patients with a history of cancer, past or present, whose tumor had recurred, (2) patients with cognitive and/or neurological deficits and physical limitations that could interfere with sexual function. Patients or the public were not involved in the design, or conduct, or reporting, or dissemination plans of our research.

The control group consisted of older women without cervical cancer and lacking gynecological pathology with the same sociodemographic characteristics chosen in the study.

### 2.3. Data Collection

Data were collected by self-administration of formatted questionnaires. First, basic demographic data (sex, age, education, sexual orientation, among others) were collected after obtaining informed consent. Sexual activity-related issues were also assessed, such as the onset of coital sexual activity, number of partners or sexual activity before and after treatment, which were collected with an ad-hoc questionnaire. The control variables were assessed through the patient's clinical history, with informed consent for access to medical data, which included the following items: histological type of tumor, cancer staging and type of treatment performed, time elapsed between the end of treatment and the study.

The following questionnaires were provided:

- Female Sexual Function Index (FSFI). The Spanish version of the questionnaire [18] is a multidimensional self-report instrument to assess female sexual function during the last 4 weeks. The questionnaire contains 19 items distributed in six subscales: sexual desire (items 1 and 2), arousal (items 3, 4, 5 and 6), lubrication (items 7, 8, 9 and 10), orgasm (items 11, 12 and 13), satisfaction (items 14, 15 and 16), and pain (items 17, 18 and 19). The range of scores for items 3 to 14 and 17 to 19 is between 0 and 5 and for items 1, 2, 15 and for item 16 between 1 and 5. The overall scale score ranges from 2 to 36, with higher scores indicating better sexual function. Conversely, low scores indicate multiple problems related to sexual function. The diagnosis of sexual dysfunction is established with global scores less than or equal to 26. The internal consistency of the questionnaire was optimal, with a Cronbach's α of 0.97.
- Golombok Rust Inventory of Sexual Satisfaction (GRISS) or sexual satisfaction inventory. The Spanish version of the questionnaire [19] was used to assess the patient's sexual satisfaction. The questionnaire assesses nine dimensions (noncommunication, infrequency, dissatisfaction, avoidance, nonsensuality, vaginismus, anorgasmia, erectile dysfunction and premature ejaculation) in both men and women, although in the present study only women and not their male partners were assessed. A score of 5 or more points in any category indicates sexual dissatisfaction. The main advantage of the GRISS questionnaire is the large number of domains assessed, which makes it possible to better describe the problems of the couple. These problems are both sexual and relationship problems (communication within the couple, satisfaction with the sexual relationship, avoidance of sexual encounters, use of erotic games or frequency of sexual intercourse). The internal consistency of the questionnaire measured by Cronbach's α was 0.82.
- European Organization for Research and Treatment of Cancer quality of life questionnaire (EORTC QLQ-30). The Spanish version of the questionnaire [20] was used to assess quality of life in different types of cancer. It consists of 30 questions assessing 15 scales: 5 functional scales (physical, emotional, social, role and cognitive function), 1 general health/QoL scale, 3 symptom scales (fatigue, pain and nausea and vomiting), as well as several aspects and symptoms common in cancer patients (dyspnea, insomnia, loss of appetite, constipation, diarrhea and financial difficulties). For each question, a score from 1 to 4 is assigned, except for the general health subscale, which is scored from 1 to 7. In terms of interpretation, the results are transformed into scores from 0 to 100. A high score, greater than 75, indicates a better perception of quality of life while those patients with values less than or equal to 75 are classified as patients with low quality of life. For the five functional scales and the general quality of life scale a high score means a good level of functionality. For the symptom scales, high scores are related to higher symptom severity. The internal consistency of the questionnaire was elevated, with a Cronbach's α of 0.85.

### 2.4. Data Analysis

Data were analyzed using SPSS version 26 software (IBM Corporation, Armonk, NY, USA). Descriptive statistics were used to calculate means and standard deviations for quantitative variables, and percentage for categorical variables. The distribution of data was analyzed using the Shapiro–Wilk test. The nonparametric Mann–Whitney test was performed to evaluate differences between groups. Cronbach's alpha coefficient was calculated to estimate the internal consistency of each questionnaire and was "excellent" if α ≥ 0.90. *p*-value < 0.05 were considered significant. 

## 3. Results

The present study included 66 patients, 33 with cervical cancer and 33 in the control group. Sociodemographic data and those related to sexual activity are detailed and compared in Table 1. There were no significant differences between the groups in the variables studied. The profile of most of the participants was 42−49 years old (50.0%) with a high level of education (39.39%). Fifty-nine percent of the participants were married; 80.3% of the participants reported having satisfactory relationships without difficulties.

Regarding the clinical characteristics of the participants, the main treatment consisted of a combination of chemotherapy, radiotherapy and brachytherapy (60.6%). The time between the end of treatment and response to the questionnaires was mostly between 6 months and 1 year (36.4%) (data not shown).

Table 2 shows the FSFI scores. The internal consistency of the questionnaire was excellent, with a Cronbach's α of 0.97. Significant differences were observed in all domains assessed. The diagnosis of sexual dysfunction was established with an overall score of 26 or less. Accordingly, the cervical cancer survivor group obtained an overall mean score of 14.22±9.61, demonstrating sexual dysfunction. However, the control group had a score of 29.00±6.66, meaning that they did not have sexual dysfunction. In addition, subscale scores (desire, excitement, lubrication, orgasm, satisfaction and pain) were found to be significantly decreased in the patient group compared to the control group (*p* < 0.05).

The GRISS questionnaire scores have shown in Table 3. The internal consistency of the questionnaire was high, with a Cronbach's α of 0.82. Significant differences were observed between the two groups in all domains. Sexual dysfunction is observed in all domains (*p* < 0.05) in the cervical cancer survivor group, except for the no communication subscale. In the control group, there is no evidence of sexual dysfunction in any domain (*p* > 0.05).

The analysis of the EORTC QLQ-30 questionnaire is shown in Table 4. The internal consistency of the questionnaire was high, with a Cronbach's α of 0.85. Cervical cancer survivors had physical (39.39 ± 25.87), emotional (62.37 ± 27.33), social (63.13 ± 33.78) and economic (18.18 ± 30.15) difficulties, with role operation (87.87 ± 22.92) and cognitive functioning (78.28 ± 24.11) being the least affected. However, only physical functioning (25.75 ± 19.36) was affected in the control group. Significant differences (*p* < 0.05) were observed between the control group and patient group in all role functioning scales except cognitive functioning.

Regarding the symptoms scale, scores in the pain (51.51 ± 38.27) and diarrhea (16.16 ± 23.74) domains were significantly increased compared to the control group (*p* < 0.05). 

For the interpretation of global health status in cervical cancer survivors, the results were transformed into a score from 0 to 100, where a high score indicated a better global health status. The analysis revealed that significant differences were found in global health status (*p* < 0.05). The overall health status in cervical cancer survivors has a mean of 60.61 ± 25.88, while in the control group the score was 74.24 ± 19.36.

## 4. Discussion

The purpose of the present study was to evaluate and compare quality of life and sexual functioning and satisfaction between cervical cancer survivors and healthy women in a Spanish population. Although incidence and mortality rates associated with cervical cancer have declined due to vaccines and prevention policies [21], the persistence of disabling sequelae is high. Therefore, the identification of this dysfunction is vital to identifying problems and improving the quality of life of cervical cancer survivors. For this purpose, a retrospective case–control study of women sexually active in the last 6 months was carried out.

The results of our study show that the quality of life and sexual function of cervical cancer survivors were worse in almost all parameters analyzed compared to the control group. In fact, according to our data, cervical cancer survivors suffer from sexual dysfunction as measured by the FSFI, while the control group showed no sexual dysfunction. Our results are consistent with previous findings in the literature [7,22]. Thus, in the work of Abd and colleagues (2021), women had sexual dysfunction and high scores in all domains of the FSFI [23]. Similarly, Harding and colleagues (2014) found that desire, arousal, lubrication, orgasm and satisfaction were affected in the group of cervical cancer survivors after radiotherapy [7]. 

To our knowledge, this is the first study to analyze the sexual function and satisfaction of women diagnosed with cervical cancer after 6 months of medical–surgical treatment in a Spanish population. However, similar results have been obtained in Latin American populations, where the effects of the disease and treatment that compromise the quality of sexual life of women with cervical cancer have been identified [24]. Nevertheless, none of them compared the results with a control group. In fact, a recent review indicates that there is a lack of research on sexual dysfunction in cervical cancer survivors due to study limitations, such as inconsistent comparison groups and lack of use of standardized questionnaires [5].

It is important to note that sexual dysfunction may be due to the type of treatment. Sexual and reproductive side effects of cervical cancer treatments include reduced sexual desire, lack of arousal and orgasm, decreased lubrication and sensation, premature menopause, loss of fertility, reduced vaginal elasticity, shortening of the vaginal cavity, vaginal atrophy and stenosis, and cystitis, which often lead to sexual dysfunction [25]. Thus, in our study, 60.6% of the women had received mixed treatment (chemotherapy, radiotherapy and brachytherapy). Other researchers have indicated that such combined treatment may be related to the stage of the cancer and, therefore, may seriously aggravate vaginal involvement and sexual function [24]. Shankar and colleagues (2020) concluded that combined treatment (surgery and radiotherapy) causes more sexual dysfunction and dissatisfaction than the use of a single specific technique to treat their disease [26]. 

Cervical cancer survivors showed worse quality of life than controls in most domains measured by the EORT QLQ-30. Financial difficulties, physical, emotional and social functioning were the most affected functional difficulties. Pain, fatigue, insomnia and appetite were the most problematic symptomatology in cervical cancer survivors. These findings are consistent with others in the literature showing poorer quality of life in cervical cancer patients [27]. In addition, patients with cervical cancer treated with NACT and subsequent radical hysterectomy, rather than standard therapies, showed poor quality of life and sexual function compared to healthy controls [3]. However, other research show no significant differences with healthy controls in the quality of life and sexual function of ovarian cancer survivors, although social functioning and financial status deteriorate [28].

Therefore, our data highlight the urgent need to develop direct personalized therapeutic treatments to reduce sexual dysfunction and, consequently, preserve quality of life in cervical cancer survivors. In addition, it would be interesting to assess whether sexual counselling and health education prior to medical–surgical treatment could reduce sexual dysfunction and dissatisfaction after treatment. 

The main limitation of this work is the sample size, which makes it difficult to obtain statistically significant comparisons to generalize the results to the population. Furthermore, the women were evaluated only after they had completed treatment for cervical cancer. Therefore, there is the possibility of pretreatment differences. In addition, the lack of longitudinal data prevents us from drawing conclusions about changes in sexual health over time. A prospective longitudinal design with a pretreatment baseline would address this important limitation.

## 5. Conclusions

Cervical cancer survivors showed worse sexuality and quality of life than the control group. Our results indicate that there is dysfunction, sexual dissatisfaction and a lower level of quality of life in cervical cancer survivors than in healthy women without pathology. These findings contribute to establish new goals and adapt therapeutic strategies in cervical cancer survivors, addressing gynecological symptoms early after treatment and advocating for an active and dysfunction-free sex life. Therefore, our data highlight the importance of continuing, once the cancer has been successfully treated, to follow up patients in the sexual sphere from the point of view of care. However, due to the small sample size, further studies with a Spanish population are needed to confirm the findings of the study.

## Figures and Tables

**Table 1:** Sociodemographic data and variables related to sexual activity in both groups.

Variable		Patient Group (*n* = 33) *n* (%)	Control Group (*n* = 33) *n* (%)	Value	*p*
Age (years)	26−33	1 (3.03)	2 (6.08)	t = −0.44	0.66
	34−41	8 (24.24)	2 (6.08)		
	42−49	14 (42.43)	19 (57.56)		
	50−57	9 (27.27)	10 (30.28)		
	58−65	1 (3.03)	0 (0)		
Educational level	No education	2 (6.08)	1 (3.03)	t = −1.37	0.17
	Primary	9 (27.27)	4 (12.12)		
	Secondary	9 (27.27)	15 (45.47)		
	Higher	13 (39.38)	13 (39.38)		
Current marital status	Single (12)	4 (12.12)	0 (0)	t = −0.47	
	Couple (18)	6 (18.18)	10 (30.28)		
	Married (55)	18 (54.55)	21 (63.63)		0.64
	Separated/divorced (12)	4 (12.12)	2 (6.09)		
	Widow (3)	1 (3.03)	0 (0)		
Sexual activity	Satisfactory relationships without difficulty	27 (81.80)	26 (78.75)		
	Unsatisfactory relationships in general	1 (3.03)	2 (6.08)	t = 0.14	0.87
	Painful intercourse	1 (3.03)	1 (3.03)		
	Problems with sexual desire	2 (6.08)	1 (3.03)		
	Satisfactory relationships and problems with sexual desire	1 (3.03)	2 (6.08)		
	Unsatisfactory relationships, painful problems with orgasm desire and arousal	1 (3.03)	1 (3.03)		

**Table 2:** FSFI scores.

Variable	Patient Group (*n* = 33) Score (Mean ± SD)	Control Group (*n* = 33) Score (Mean ± SD)	U-Mann Whitney	*p*
FSFI global score	14.22 ± 9.61	29.00 ± 6.66	115.50	<0.001
Desire	2.54 ± 1.43	3.89 ± 1.35	267.00	<0.001
Excitement	2.34 ± 1.81	5.03 ± 1.25	110.00	<0.001
Lubrication	1.95 ± 2.04	4.92 ± 1.49	156.00	<0.001
Orgasm	2.48 ± 2.15	5.23 ± 1.20	136.00	<0.001
Satisfaction	2.95 ± 1.62	4.77 ± 1.44	217.50	<0.001
Pain	1.93 ± 2.01	5.13 ± 1.74	121.50	<0.001

FSFI: Female Sexual Function Index.

**Table 3:** GRISS scores.

Variable	Patient Group (*n* = 33) Score (Mean ± SD)	Control Group (*n* = 33) Score (Mean ± SD)	Mann–Whitney-U	*p*
No communication	4.87 ± 2.72	3.43 ± 1.93	361.50	0.07
Infrequency	6.42 ± 2.46	5.00 ± 1.98	331.50	0.01
Dissatisfaction	4.33 ± 1.83	2.75 ± 1.74	258.00	<0.001
Not sensuality	2.21 ± 1.99	1.18 ± 0.73	333.00	0.001
Avoidance	6.00 ± 2.69	3.71 ± 2.47	287.00	0.001
Anorgasmia	3.81 ± 1.48	2.43 ± 1.21	245.50	<0.001
Vaginismus	7.30 ± 1.15	3.81 ± 2.10	59.00	<0.001

GRISS: Golombok Rust Inventory of Sexual Satisfaction.

**Table 4:** EORTC QLQ-30 scores.

**Functional Scale**				
**EORT QLQ-30**	**Patient Group (*n* = 33)** **Score (Mean ± SD)**	**Control Group (*n* = 33)** **Score (Mean ± SD)**	**Mann–Whitney-U**	***p***
Physical functioning	39.39 ± 25.87	25.75 ± 19.36	366.00	0.02
Role operation	87.87 ± 22.92	99.49 ± 2.90	361.00	<0.001
Emotional functioning	62.37 ± 27.33	75.25 ± 21.89	385.50	0.04
Cognitive functioning	78.28 ± 24.11	85.85 ± 19.14	449.00	0.19
Social functioning	63.13 ± 33.78	83.33 ± 21.65	351.50	0.01
Financial difficulties	18.18 ± 30.15	30.15 ± 16.15	385.00	0.004
**Symptoms Scale**				
**EORT QLQ-30**	**Patient Group (*n* = 33)** **Score (Mean ± SD)**	**Control Group (*n* = 33)** **Score (Mean ± SD)**	**Mann–Whitney-U**	***p***
Fatigue	34.34 ± 28.51	25.25 ±21.74	451.50	0.23
Vomiting	24.24 ± 26.05	13.63 ± 17.40	414.00	0.08
Pain	51.51 ± 38.27	35.35 ± 8.07	425.00	0.02
Dyspnea	12.12 ± 21.75	6.25 ± 13.21	474.00	0.33
Insomnia	33.33 ± 26.35	24.24 ± 25.37	441.00	0.16
Diarrhea	16.16 ± 23.74	6.06 ± 14.48	425.00	0.04
Appetite	33.33 ± 26.35	24.24 ± 25.37	441.00	0.16
Constipation	19.19 ± 22.09	18.18 ± 27.75	502.00	0.54
**Global Health Status Scale**				
**EORT QLQ-30**	**Patient Group (*n* = 33)** **Score (Mean ± SD)**	**Control Group (*n* = 33)** **Score (Mean ± SD)**	**Mann–Whitney-U**	***p***
Global health status	60.61 ± 25.88	74.24 ± 19.36	366.00	0.02

EORT QLQ-30: European Organization for Research and Treatment of Cancer quality of life questionnaire.

## Data Availability

The data that support the findings of this study are available on request from the corresponding author.
